# A novel lncRNA-miRNA-mRNA triple network identifies lncRNA XIST as a biomarker for acute myocardial infarction

**DOI:** 10.18632/aging.204075

**Published:** 2022-05-10

**Authors:** Peng-Fei Zheng, Lu-Zhu Chen, Peng Liu, Hong-Wei Pan

**Affiliations:** 1 Cardiology Department, Hunan Provincial People's Hospital, Furong District, Changsha 410000, Hunan, China; 2 Clinical Research Center for Heart Failure in Hunan Province, Furong District, Changsha 410000, Hunan, China; 3 Institute of Cardiovascular Epidemiology, Hunan Provincial People's Hospital, Furong District, Changsha 410000, Hunan, China; 4 Department of Cardiology, The Central Hospital of ShaoYang, Daxiang District, Shaoyang 422000, Hunan, China

**Keywords:** weighted gene co-expression network analysis, acute myocardial infarction, significant modules, hub genes, lncRNA-miRNA-mRNA network

## Abstract

Despite the well-established role of long non-coding RNAs (lncRNAs) across various biological processes, their mechanisms in acute myocardial infarction (AMI) are not fully elucidated. The GSE34198 dataset from the Gene Expression Omnibus (GEO) database, which comprised 49 specimens from individuals with AMI and 47 specimens from controls, was extracted and analysed using the weighted gene co-expression network analysis (WGCNA) package. Twenty-seven key genes were identified through a combination of the degree and gene significance (GS) values, and the *CDC42* (degree = 64), *JAK2* (degree = 41), and *CHUK* (degree = 30) genes were identified as having the top three-degree values among the 27 genes. Potential interactions between lncRNA, miRNAs and mRNAs were predicted using the starBase V3.0 database, and a lncRNA-miRNA-mRNA triple network containing the lncRNA *XIST*, twenty-one miRNAs and three hub genes (*CDC42*, *JAK2* and *CHUK*) was identified. RT–qPCR validation showed that the expression of the *JAK2* and *CDC42* genes and the lncRNA *XIST* was noticeably increased in samples from patients with AMI compared to normal samples. Pearson’s correlation analysis also proved that *JAK2* and *CDC42* expression levels correlated positively with lncRNA *XIST* expression levels. The area under ROC curve (AUC) of lncRNA *XIST* was 0.886, and the diagnostic efficacy of the lncRNA *XIST* was significantly better than that of *JAK2* and *CDC42*. The results suggested that the lncRNA *XIST* appears to be a risk factor for AMI likely through its ability to regulate *JAK2* and *CDC42* gene expressions, and it is expected to be a novel and reliable biomarker for the diagnosis of AMI.

## INTRODUCTION

Coronary artery disease (CAD) is a ubiquitous chronic heart disease involving the deposition and build-up of atherosclerotic plaques in the coronary artery, leading to a gradual constriction of the vascular lumen and compromised myocardial perfusion [1]. CAD manifests in several different ways, including acute myocardial infarction (AMI), unstable and stable angina, ischemic cardiomyopathy and even sudden cardiac death [2]. The advent of emergency percutaneous coronary intervention (PCI), which rapidly restores cardiac perfusion, has resulted in a tremendously improved prognosis for patients with AMI. Nevertheless, AMI is still the main cause of death in patients with CAD worldwide. AMI is responsible for significant patient morbidity and mortality, especially in China, where these rates have been increasing annually [3, 4]. Epidemiological studies related to AMI have shown that male sex, hyperlipidaemia, smoking, stress, diabetes, hypertension, age, obesity, family history and a sedentary lifestyle all culminate in the development of AMI [5]. Recent research has focused on identifying gene-based prognostic and therapeutic markers for AMI to circumvent the daunting challenge of managing patients with AMI. Interest in gene sequencing technology has increased, because it allows clinicians to obtain deeper insights into the relationship between genes and diseases [6, 7].

Several compelling studies have proposed that several noncoding RNAs (ncRNAs) are intricately involved in multiple biological functions and possess critical functions in the occurrence and development of diseases [8, 9]. Based on transcript length, noncoding RNAs are functionally grouped into two specific subtypes: (1) long ncRNAs (lncRNAs) (> 200 nt), which comprise long intergenic ncRNAs (lincRNAs), natural antisense transcripts, transcribed ultra-conserved regions, and enhancer-like ncRNAs; and (2) short ncRNAs (< 200 nt), which comprise PIWI-interacting RNAs, microRNAs, and transcription initiation RNAs [10, 11]. Unlike highly conserved short ncRNAs, whose function is essentially to participate in posttranscriptional modification, lncRNAs are less well conserved but are implicated in several physiological processes [12, 13].

Recent studies have proposed the essential role of lncRNAs in cardiovascular diseases [14]. Wang et al. confirmed that a lncRNA cardiac and apoptosis-related RNA (CARL), which has previously been associated with cardiomyocyte apoptosis, functions as a miR-539 molecular sponge, leading to inhibited mitochondrial division and apoptosis by stimulating *PHB2* gene function [15]. In addition, the lncRNA wisp2 super enhancer related RNA (Wisper) stimulates myocardial fibrosis after myocardial infarction [16]. More importantly, several regulatory networks between lncRNAs and microRNAs (miRNAs) have recently been documented, providing insights into the exact functions of noncoding RNAs and their potential as molecular targets when developing therapeutic modalities for certain illnesses [17, 18]. Therefore, in this study, a scale-free network was constructed using weighted gene co-expression network analysis (WGCNA) [19], followed by a modularized analysis on the scale-free network and in-depth scrutinization of the correlations between modules, phenotypes and clinical data. Finally, hub genes requiring further study were identified among the genes that were significantly associated with phenotypes present in the meaningful modules. Furthermore, using data extracted from the starBase V3.0 database, we examined several miRNAs and lncRNAs that may target the identified hub genes and constructed a lncRNA-miRNA-mRNA triple network to identify specific lncRNAs that may have potential as sensitive and specific AMI biomarkers.

## RESULTS

### Data preprocessing

Data were first processed by standardizing data formats, adding missing values and removing outliers. A total of 24580 gene symbols were detected in 97 samples. The co-expression network was constructed by selecting the top 25% of genes (6145) with a large variance in expression levels. Gene expression profiles of 6145 genes and clinical characteristics of the samples are described in detail in Supplementary Tables 2, 3.

### Weighted gene co-expression networks

**Figure 1:**
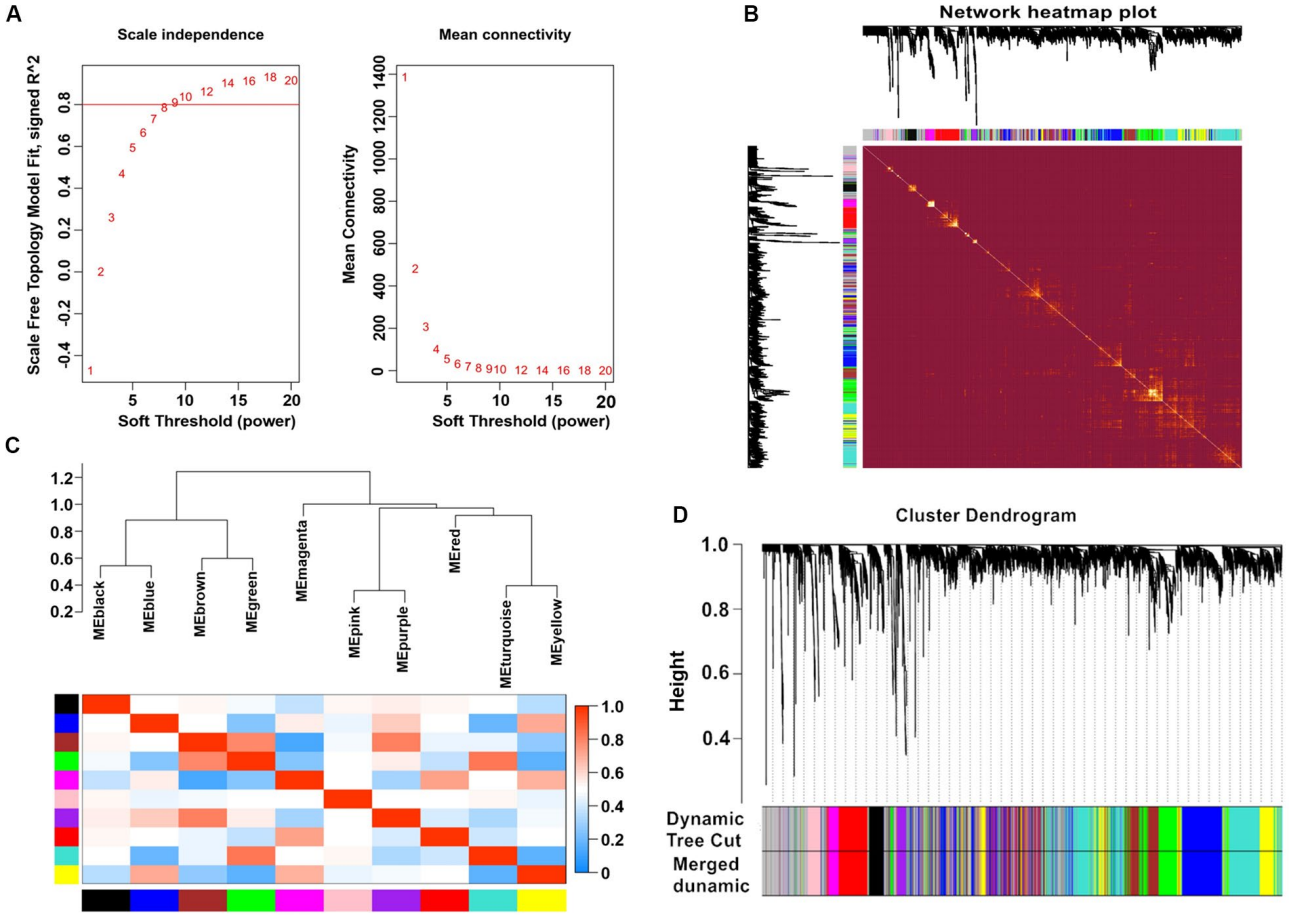
**Weighted gene co-expression network analysis.** (**A**) Analysis of network topology for various soft-thresholding powers. (**B**) Heatmap of the topological overlap in the gene network. (**C**) Relationship among all the modules. (**D**) Clustering dendrogram of genes. Gene clustering tree (dendrogram) obtained by hierarchical clustering of adjacency-based dissimilarity.

After calculation, we discovered that a correlation coefficient greater than 0.8 (the soft threshold of β is 10) was highly correlated and suitable for the construction of various gene modules (Figure 1A). A topological overlap matrix (TOM) was constructed by calculating the correlation and adjacency matrices of gene expression profiles. The gene cluster tree is depicted in Figure 1B. We then sought to identify the gene modules of each gene network using the hierarchical average linkage clustering method combined with TOM. Figure 1C depicts the heatmap. The dynamic tree cut algorithm identified ten gene modules (Figure 1D).

### Determining modules of interest

**Figure 2:**
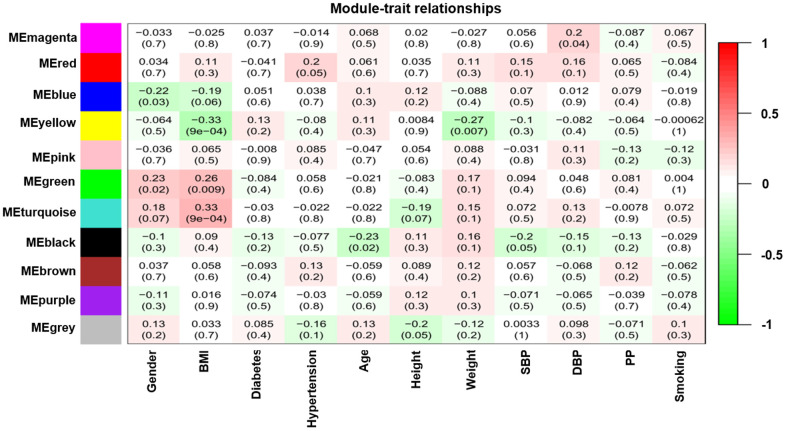
**Module-feature associations.** Each row corresponds to a modulEigengene and the column to the clinical phenotype. Each cell contains the corresponding correlation in the first line and the P-value in the second line. The table is color-coded by correlation according to the color legend.

**Figure 3:**
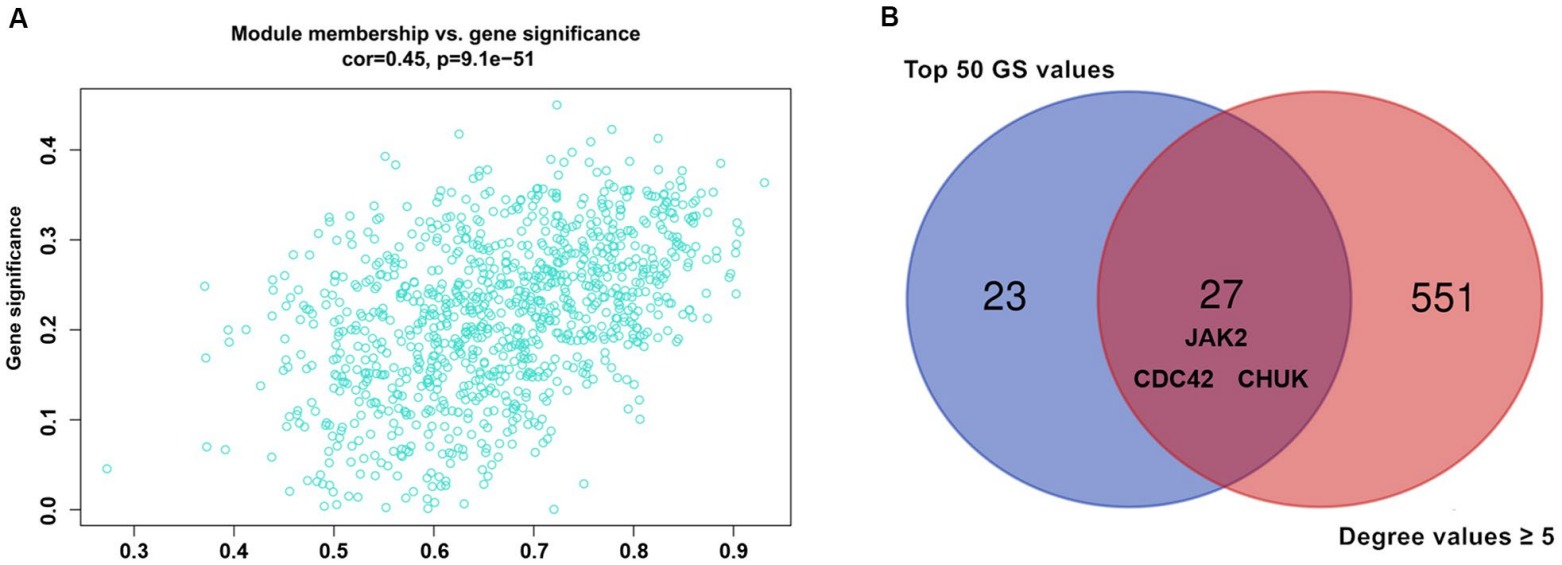
(**A**) Association between gene significance and module membership. Scatterplot shows a highly significant correlation between gene significant (GS) versus module membership (MM) with AMI in the turquoise module. (**B**) key genes with top 50 GS values and they degree values ≥ 5 were defined by Venn diagram.

Modules closely related to clinical features often have important and specific biological significance. As shown in Figure 2, the turquoise module appeared to be highly correlated with BMI (*r ^2^* = 0.33, *P* = 9e-04). An in-depth calculation was performed to discern the association between the colour module and gene significance (GS). Figure 3A shows that the association between the turquoise module and gene significance was 0.45 (*P* = 9.1E-51). All gene symbols in the turquoise module and their GS values and corresponding *P* values are described in detail in Supplementary Table 4.

### Module preservation test

Gene expression profiles of patients with AMI were subjected to a preservation analysis. We identified one strong module and two moderately preserved modules between patients with AMI and control subjects (Supplementary Figure 1). Both Zsummary and medianRank statistical results were consistent, suggesting that the preservation analysis was not affected by the size of the module. The turquoise module was noted to be highly preserved, while the yellow and blue modules were moderately preserved. These interesting findings suggested the presence of significant variabilities in the gene expression patterns between the AMI and control groups.

### Enrichment analysis of interesting modules

**Figure 4:**
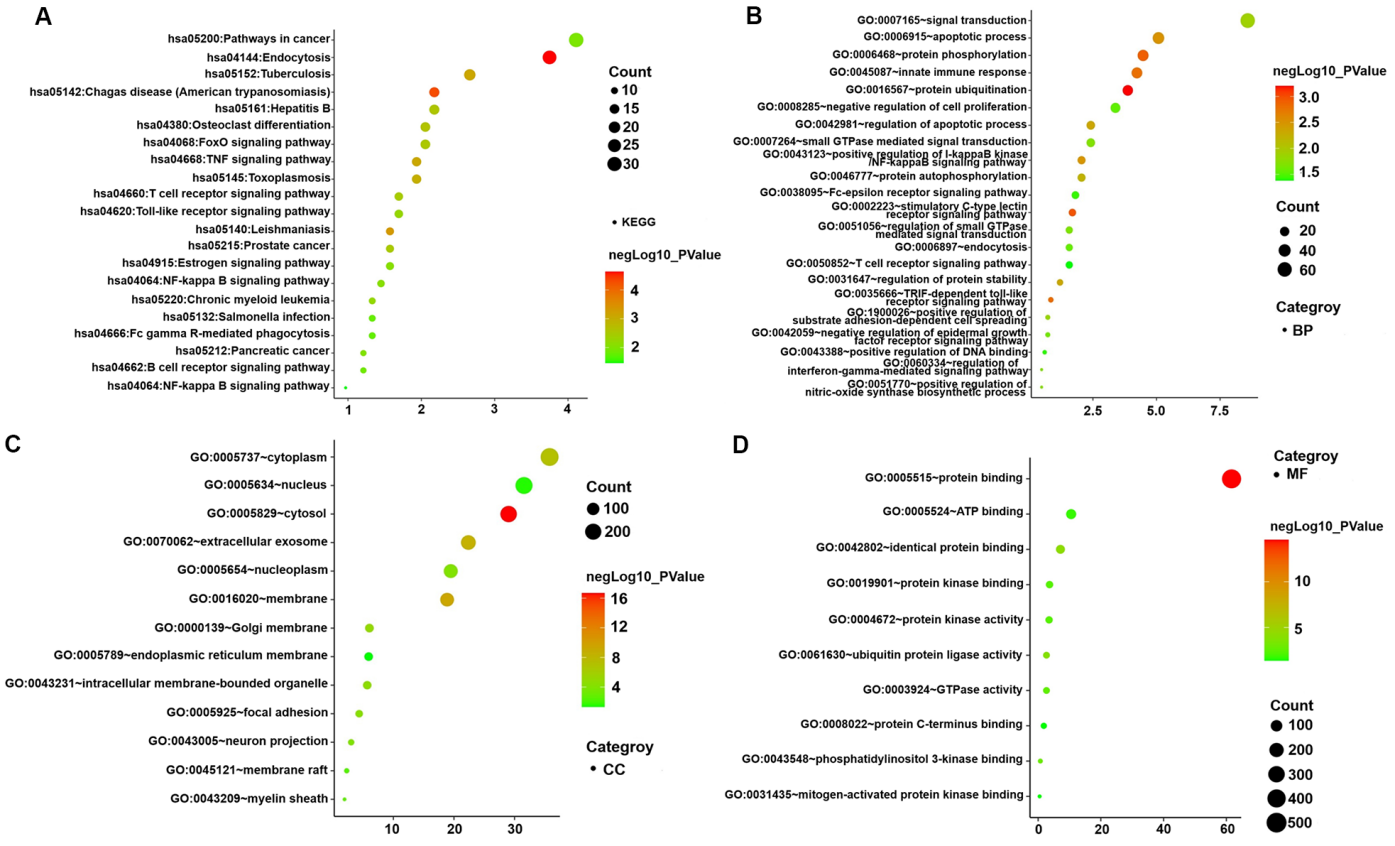
**GO functional and KEGG pathway enrichment analyses for genes in the object module.** The x-axis shows the number of genes and the y-axis shows the GO and KEGG pathway terms. The -log10 (P-value) of each term is colored according to the legend. (**A**) KEGG pathway. (**B**) Biological process. (**C**) Cytological component. (**D**) Molecular function.

KEGG pathway and GO enrichment analyses of genes in the turquoise module were performed to dissect their physiological purposes functions. Figure 4A depicts the KEGG signalling pathways, and the biological process, cytological component and molecular function are shown in Figure 4B–4D. Nine hundred ninety-five genes in the turquoise module were primarily enriched in the following potentially AMI-related inflammatory pathways: hsa05152: tuberculosis; hsa04668: TNF signalling pathway; hsa05161: hepatitis B; hsa04068: FoxO signalling pathway; hsa04660: T cell receptor signalling pathway; hsa04620: Toll-like receptor signalling pathway; hsa04064: NF-kappa B signalling pathway; hsa04662: B cell receptor signalling pathway; hsa05132: *Salmonella* infection; hsa04666: Fc gamma R-mediated phagocytosis; hsa04932: nonalcoholic fatty liver disease; hsa05164: influenza A; and hsa04621: NOD-like receptor signalling pathway. The details of these analyses are also presented in Supplementary Tables 5, 6.

### Construction of a PPI network and identification of hub genes

**Figure 5:**
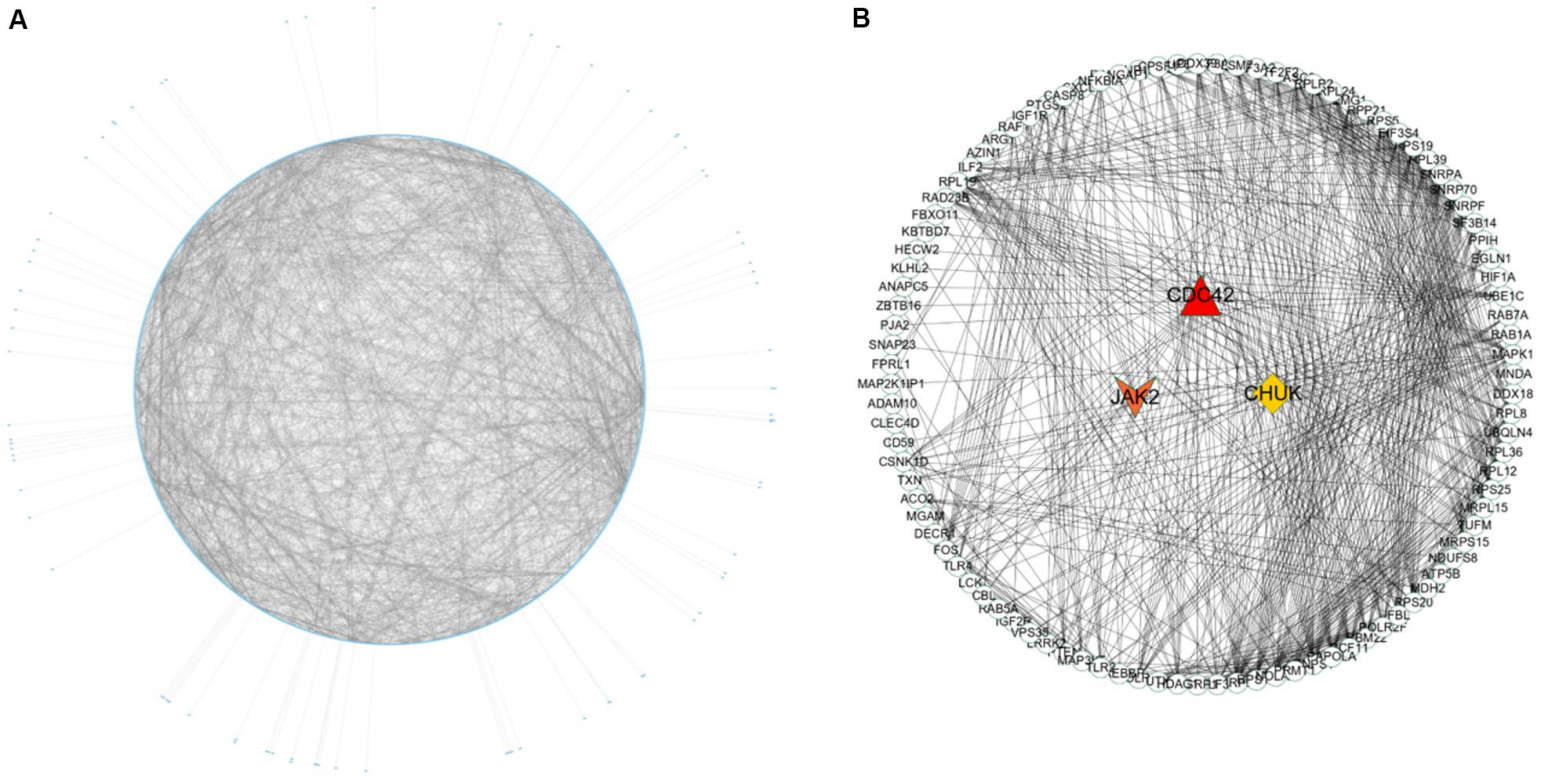
**PPI network construction and identification of hub genes.** (**A**) PPI network of genes in turquoise module. The edge shows the interaction between two genes. (**B**) PPI network of genes with top 100-degree values.

Nine hundred ninety-five genes in the turquoise module were used to build the PPI network. As shown in Figure 5A, a PPI network consisting of 936 nodes and 4107 edges was built using the STRING tool. In addition, a PPI network of genes with the top 100-degree values is depicted in Figure 5B to better show the interaction between key genes and other genes. We noticed that the *JAK2*, *CDC42* and *CHUK* genes are widely associated with other genes. The gene degree values were calculated using the Cytohubba plug-in in Cytoscape software, and gene degree values ≥ 5 are also shown in Supplementary Table 7. As shown in Figure 3B and Supplementary Table 8, 27 genes with the top 50 GS values and degree values ≥ 5 were defined as key genes, and the *CDC42* (degree = 64), *JAK2* (degree = 41), and *CHUK* (degree = 30) genes were identified as having the top three-degree values among the 27 genes. Then, combined with GO and KEGG analysis results, we noticed that the *JAK2*, *CDC42* and *CHUK* genes are involved in the signalling pathways and biological processes related to AMI; therefore, the *JAK2*, *CDC42* and *CHUK* genes were selected as hub genes for further research.

### Construction of the lncRNA-miRNA-mRNA regulatory network

**Figure 6:**
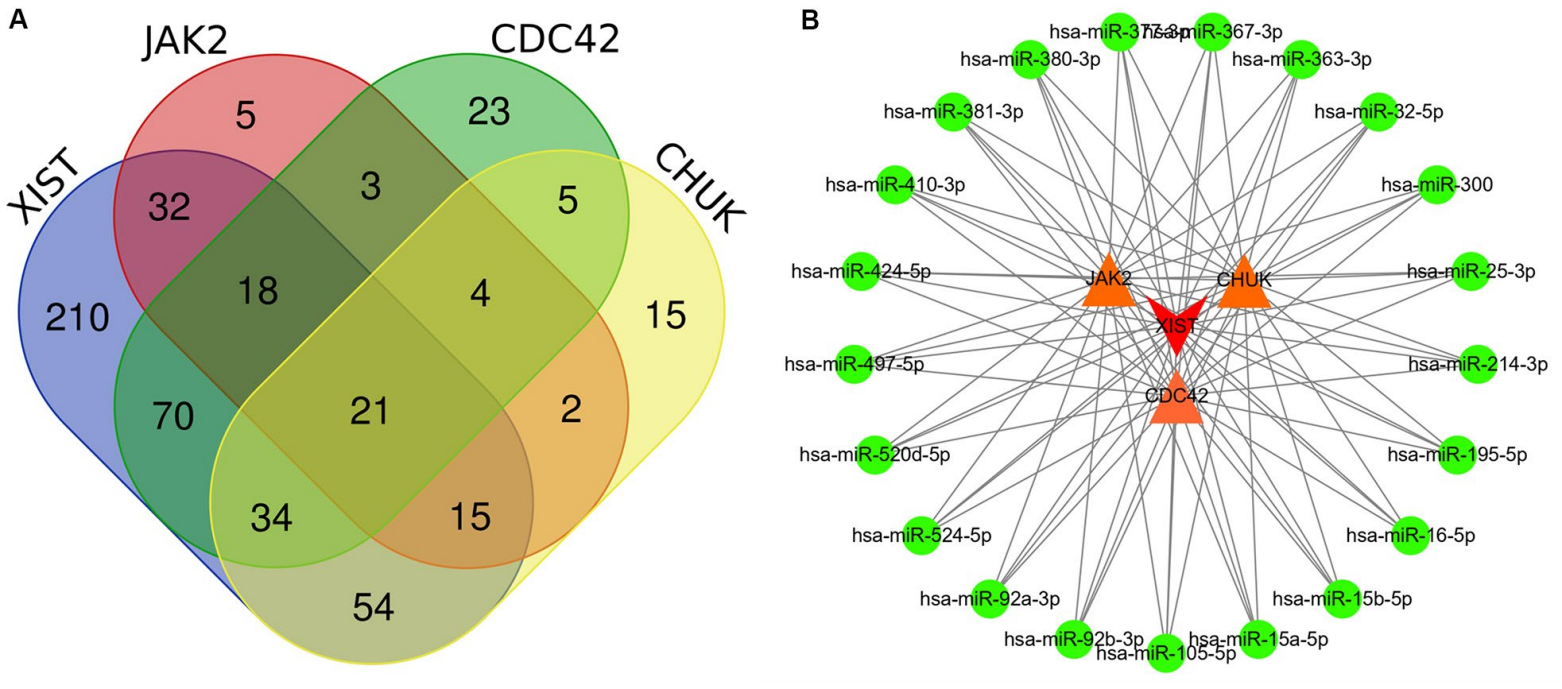
(**A**) Several common miRNAs that target *JAK2*, *CDC42*, and *CHUK* genes and lncRNA *XIST* were identified by Venn diagram. (**B**) A lncRNA-miRNA-mRNA ceRNA network that contained one lncRNA *XIST*, 21 miRNAs and 3 mRNAs (*JAK2*, *CDC42* and *CHUK*). Edge stands for the interaction between two items.

First, we identified lncRNAs that may potentially target the *JAK2*, *CDC42* and *CHUK* genes based on information obtained from the starBase database. As shown in Supplementary Table 9, only one lncRNA, *XIST* was identified that might represent a common lncRNA forming ceRNA networks with both the *JAK2* and *CDC42* genes. As shown in Supplementary Tables 10, 11, we predicted miRNAs that potentially bind to the *JAK2*, *CDC42*, and *CHUK* genes and the lncRNA *XIST*. One hundred miRNAs bound to *JAK2*; 178 miRNAs bound to *CDC42*; 150 miRNAs bound to *CHUK*; and 454 miRNAs bound to the lncRNA *XIST*. As shown in Figure 6A, 21 common miRNAs (Supplementary Table 12) bound to the *JAK2*, *CDC42*, and *CHUK* genes and the lncRNA *XIST* simultaneously. Using this information, we built a lncRNA-miRNA-mRNA network that was visualized using Cytoscape software (version 3.71) (Figure 6B).

### Validation analysis using RT–qPCR and Pearson’s correlation analysis

**Figure 7:**
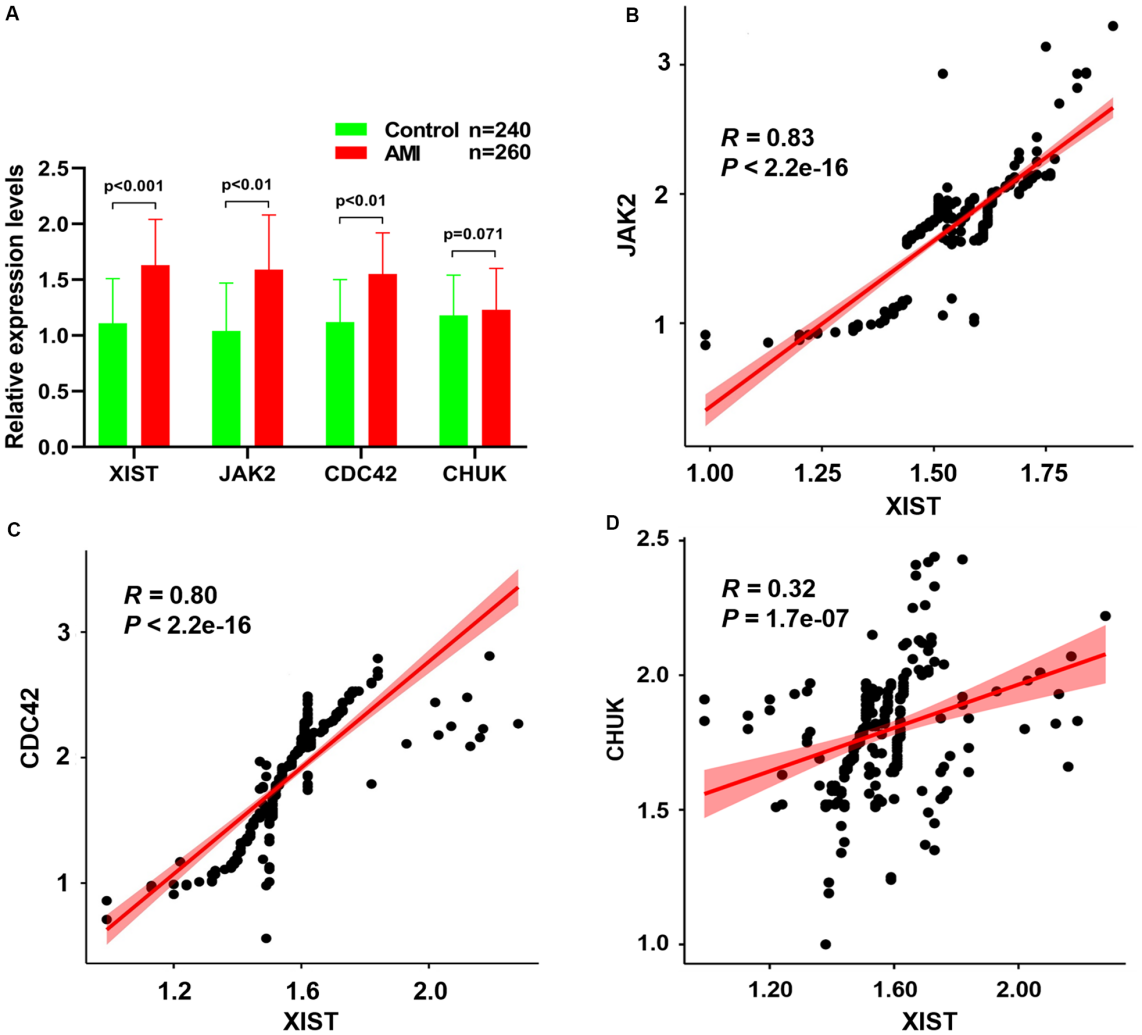
(**A**) The relative expression levels of *JAK2*, *CDC42*, *CHUK* genes and lncRNA *XIST* between healthy controls and AMI samples. The correlation between the expression levels of lncRNA *XIST* and *JAK2* (**B**), *CDC42* (**C**) and *CHUK* (**D**) genes that were analyzed by Pearson correlation analysis.

Figure 7A depicts the RT–qPCR results of the relative expression levels of the *JAK2* and *CDC42* genes and the lncRNA *XIST*, which were significantly increased in patients with AMI compared to healthy controls. Meanwhile, a Pearson correlation analysis found that the expression levels of *JAK2* (Figure 7B, *R* = 0.83, *P <* 2.2E-16) and *CDC42* (Figure 7C, *R* = 0.80, *P <* 2.2E-16) were positively correlated with the lncRNA *XIST* levels. Conversely, little association was observed between the expression levels of the *CHUK* gene (Figure 7D, *R* = 0.32, *P =* 1.7E-07) and that of the lncRNA *XIST*.

### ROC curve for patients with AMI

**Figure 8:**
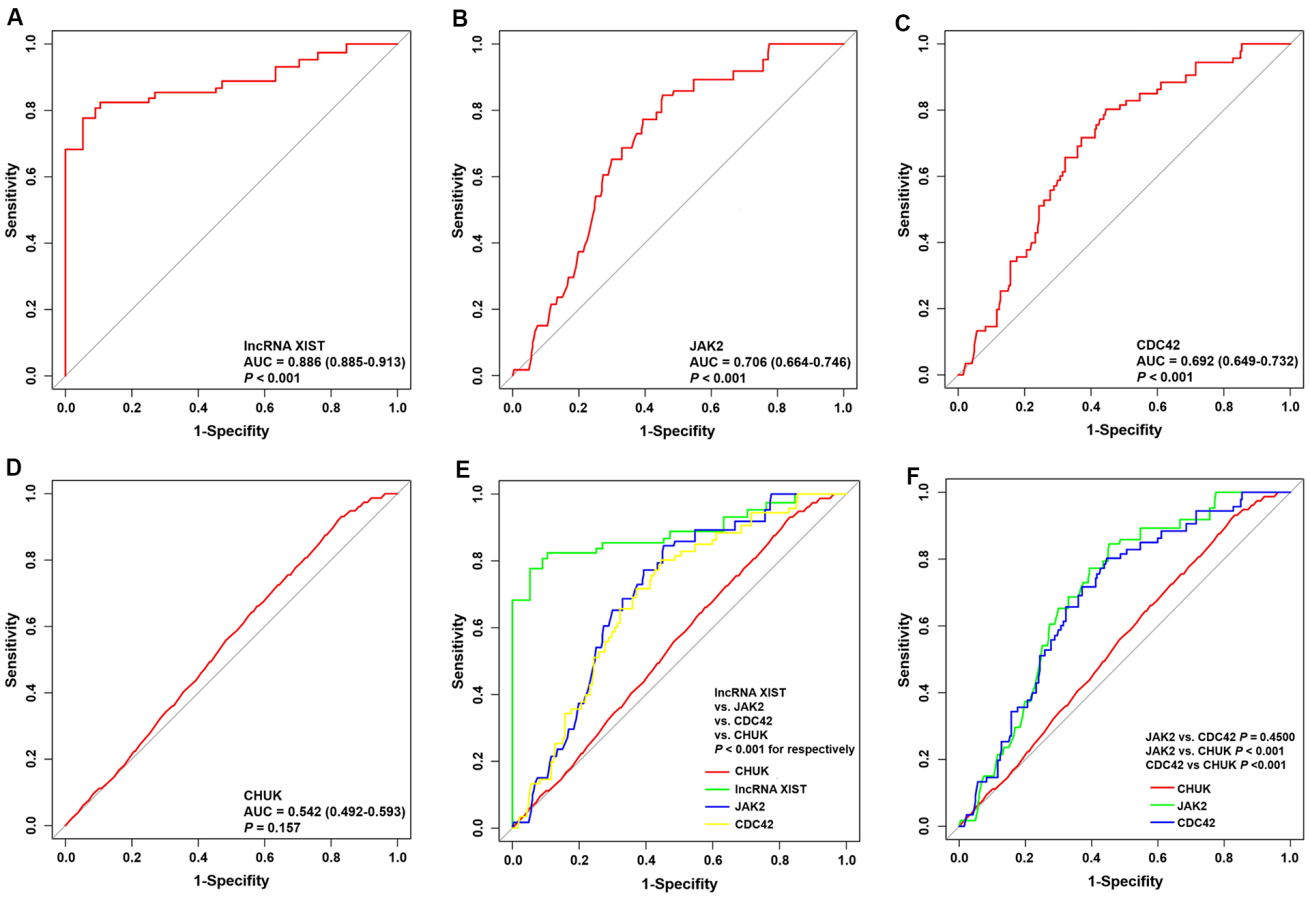
**ROC curve analyses of one lncRNA and three genes for the diagnosis of AMI.** (**A**–**D**) ROC curve analysis of lncRNA *XIST*, *JAK2*, *CDC42* and *CHUK* genes. (**E**, **F**) The pairwise *P*-value comparison.

The predictive values of the lncRNA *XIST*, *JAK2*, *CDC42* and *CHUK* for the diagnosis of AMI were investigated using a ROC curve analysis. The AUC values for the lncRNA *XIST* (Figure 8A), *JAK2* (Figure 8B), *CDC42* (Figure 8C) and *CHUK* (Figure 8D) were 0.886 (95% CI 0.885–0.913; *P* = 0.0163) with a cut-off value of 0.772, a sensitivity of 94.8% and a specificity of 82.4%, 0.706 (95% CI 0.664-0.746; *P* < 0.001) with a cut-off value of 0.560, a sensitivity of 78.7% and a specificity of 77.3%, 0.692 (95% CI 0.649–0.732; *P* < 0.001) with a cut-off value of 0.548, a sensitivity of 74.5% and a specificity of 80.3%, and 0.542 (95% CI 0.492-0.593; *P* = 0.157) with a cut-off value of 0.163, a sensitivity of 43.8% and a specificity of 72.5%, respectively. As shown in Figure 8E, 8F, we noticed that the diagnostic efficacy of the lncRNA *XIST* was significantly better than that of *JAK2*, *CDC42* and *CHUK*, and the diagnostic efficacy of *JAK2* and *CDC42* was significantly better than that of *CHUK*. However, neither *JAK2* nor *CDC42* differed significantly in terms of diagnostic efficacy.

### Demographic and biochemical characteristics

Neither patients with AMI nor individuals in the control group differed significantly in terms of weight, age, heart rate, pulse pressure, uric acid level, height, sex ratio, BMI or proportion of alcohol consumers (Table 1). Patients with AMI were more likely to smoke and have higher glucose levels, systolic and diastolic blood pressures, weight, serum low-density lipoprotein cholesterol (LDL-C), apolipoprotein (Apo) B, triglyceride (TG) and total cholesterol (TC) levels, creatinine levels, cardiac troponin T (cTnT) levels, body mass index (BMI) and creatine kinase (CK) and CK-MB levels than healthy participants. Patients in the control group had markedly increased ApoA1/ApoB ratios, ApoA1 levels and serum high-density lipoprotein cholesterol (HDL-C) levels.

**Table 1:** Comparison of demographic, lifestyle characteristics and serum lipid levels of the participants.

**Characteristic**	**Control (n=240)**	**AMI** **(n=260)**	**Test-statistic**	***P***
Male/female ^c^	176/64	187/73	0.125	0.724
Age (years) ^a^	53.7±11.72	53.45±9.15	1.931	0.486
Height (cm) ^a^	164.28±7.5	164.99±6.81	1.795	0.690
Weight (kg) ^a^	59.90±9.10	60.26±10.11	4.882	0.229
BMI (kg/m^2^) ^a^	22.31±3.84	22.09±3.34	3.498	0.352
Smoking [n (%)] ^c^	79(32.9)	108 (41.5)	3.962	0.047
Alcohol [n (%)] ^c^	64(26.7)	68(26.2)	0.017	0.897
SBP (mmHg) ^a^	131.05±19.01	136.67±22.16	9.069	0.002
DBP (mmHg) ^a^	80.01±11.99	82.90±13.35	7.193	0.018
PP (mmHg) ^a^	51.06±14.2	53.77±19.62	3.881	0.057
Glu (mmol/L) ^a^	6.05±1.57	6.45±1.76	8.646	0.009
TC (mmol/L) ^a^	4.49±1.00	4.82±1.11	11.884	2.37E-4
TG (mmol/L) ^b^	1.04(0.75)	1.43(0.64)	-2.076	0.038
HDL-C (mmol/L) ^a^	1.65±0.47	1.13±0.30	20.739	1.2035E-40
LDL-C (mmol/L) ^a^	2.78±0.98	3.06±1.01	9.033	0.004
ApoA1 (g/L) ^a^	1.42±0.33	0.98±0.29	22.261	5.7606E-43
ApoB (g/L) ^a^	0.88±0.20	0.96±0.26	17.308	1.1219E-31
ApoA1/ApoB ^a^	1.67±0.51	1.11±0.47	10.151	0.001
Heart rate (beats/minutes) ^a^	72.90±9.58	73.67±7.54	3.807	0.322
Creatinine, (μmol/L) ^a^	70.54±12.54	76.67±13.77	12.923	2.99E-7
Uric acid, (μmol/L) ^a^	270.50±74.82	280.40±78.24	5.191	0.149
Troponin T, (μg/L) ^a^	0.05±0.03	3.46±1.86	142.39	1.67E-76
CK, (U/L) ^a^	77.95±41.61	1094.10±561.61	289.76	3.23E-139
CK-MB, (U/L) ^a^	12.39±2.42	125.46±49.06	202.91	4.04E-104

SBP, Systolic blood pressure; DBP, Diastolic blood pressure; PP, Pulse pressure; Glu, Glucose; HDL-C, high-density lipoprotein cholesterol; LDL-C, low-density lipoprotein cholesterol; Apo, Apolipoprotein; TC, Total cholesterol; TG, Triglyceride; CK, creatine kinase, CK-MB, creatine kinase-myocardial band.

^a^Mean ± SD determined by t-test.

^b^Median (interquartile range) tested by the Wilcoxon-Mann-Whitney test.

^c^The rate or constituent ratio between the different groups was analyzed by the chi-square test.

## DISCUSSION

Ischemic cardiovascular disease is the primary contributor to global disability and death, despite the vast number of innovative therapeutic and diagnostic methods that have been developed in the last 10 years. In fact, studies predict a steady increase in the number of patients who will experience ischemic cardiovascular diseases, particularly AMI [20]. A reliable method of identifying early stages of AMI is needed to ensure that patients receive the best and most appropriate treatment, as well as a better prognosis. Although the measurement of traditionally available biomarkers associated with myocardial injury, such as CK-MB and cTnI/T, has significantly improved the rates and speed of AMI diagnosis, these markers are not sufficiently specific. Interestingly, AMI is characterized by a heterogeneous genetic profile, suggesting that AMI development and occurrence may rely heavily on the expression of various genes [21]. Thus, further exploration of AMI-related genetic markers may provide opportunities for the creation of more effective preventive, diagnostic and therapeutic methods for treating AMI.

A number of recent studies have shown that the occurrence of CAD results from the interaction of multiple factors, such as alterations in blood lipid levels, an unhealthy lifestyle, environmental factors and genetic background [22, 23]. As the pathological basis of CAD, atherosclerosis results from abnormal lipid metabolism and chronic inflammation [24, 25]. According to a recent study, inflammatory or immune-related genes such as intercellular adhesion molecule 1 (*ICAM1*), transmembrane immune signaling adaptor TYROBP (*TYROBP*), integrin subunit alpha M (*ITGAM*) and cathelicidin antimicrobial peptide (*CAMP*) are strongly associated with CAD [26]. Similarly, immune- and inflammation-related genes and biological processes have been reported to play crucial roles in cardiac injury and repair, and together with the activation of innate and adaptive immune responses, have been suggested to be the hallmarks of myocardial infarction (MI). As reported in previous studies, the Janus kinase/signal transducer and activator of transcription (JAK/STAT) signal pathway is required for the development of atherosclerosis [27–29]. Yang et al. reported that ruxolitinib, a JAK2-specific inhibitor, reduces the size of atherosclerotic plaques by inhibiting the JAK2/STAT3/SOCS3 signal pathway [30]. As shown in the study by Zhang et al. oncostatin M receptor β (OSMR-β) deficiency effectively delays the development of atherosclerosis and improves the stability of vulnerable plaques by suppressing the JAK2/STAT3 signal pathway, thereby reducing the incidence of AMI and ischemic stroke [31]. Moreover, Desai HR et al. also described a crucial role for *JAK2* in the pathogenesis of obesity-related inflammatory reactions and insulin resistance, and *JAK2* deficiency reduces inflammation in the liver and visceral adipose tissue in response to metabolic stress, increases insulin sensitivity and attenuates insulin resistance [32]. Geng et al. suggested that fibronectin type III domain containing 5 (FNDC5) alleviates obesity-induced cardiac hypertrophy by inhibiting JAK2/STAT3-related cardiac inflammation and oxidative stress [33]. Similarly, in the current study, we noticed that the turquoise module was strongly correlated with BMI (*r* ^2^ = 0.33, *P* = 9e-04). Meanwhile, as a key gene in the module, the expression levels of *JAK2* were significantly higher in patients suffering from AMI than in control subjects, however, whether *JAK2* could affect the pathogenesis of AMI by mediating insulin resistance and obesity remains unclear, and further experiments are needed to clarify this mechanism.

Vascular inflammation promotes the occurrence and development of atherosclerosis by accelerating the senescence of vascular endothelial cells. *CDC42*, as a member of the Rho GTPase family, plays a key role in response to pathological and physiological stimulation [34, 35]. Takashi K. Ito et al. revealed that the *CDC42* pathway contributes significantly to chronic inflammation related to endothelial cell senescence, and endothelial-specific deletion of *CDC42* markedly relieves the endothelial inflammatory response and the progression of atherosclerosis in atherosclerotic mice [36]. Raut SK et al. revealed that the miR-30c-induced increase in *CDC42* levels promotes diabetes-related myocardial injury and hypertrophy [37]. Inhibition of *CDC42* expression effectively alleviates myocardial fibrosis and hypertrophy in patients with salt-sensitive hypertension [38]. Moreover, Liu et al. revealed that *CDC42* levels are noticeably increased in the myocardium near areas of myocardial infarction, and inhibition of the expression and activity of the *CDC42* protein effectively reduces myocardial fibrosis after myocardial infarction in mice [39]. Recent studies have identified a crucial role for *CDC42* in the progression of diabetes and diabetes-associated diseases, such as insulin resistance and diabetic nephropathy [35]. Similarly, in the current study, we also noticed significantly higher *CDC42* expression in patients suffering from AMI than in control subjects. However, additional studies are needed to confirm whether *CDC42* affects the pathogenesis of AMI by mediating insulin resistance, diabetes, obesity and other processes.

Recently, lncRNAs have been repeatedly reported to be crucial modulators of the genome regulatory network and exert a significant effect on disease development [40, 41]. Transcriptome studies have noted that lncRNA expression patterns are highly cell- and tissue-specific, indicating that the disease prognosis may be predicted by assessing these patterns [42]. Previous research has described the involvement of lncRNAs in fundamental biological phenomena, including gene transcription, RNA processing, chromatin modification, gene regulation and posttranscriptional gene regulation [43]. Several meaningful lncRNAs that may exert regulatory effects on the progression of cardiovascular diseases (CVDs), such as cardiac hypertrophy, myocardial infarction and cardiovascular ageing, have been characterized [44, 45]. Zangrando et al. found that up to 30 lncRNAs are differentially expressed in AMI mouse models exhibiting left ventricular remodelling [46]. Another compelling study based on a microarray analysis identified 545 deregulated lncRNAs involved in myocardial fibrosis induced after MI [47]. Recently, lncRNA-miRNA-mRNA ceRNA networks have been utilized to explore the functional roles of lncRNAs in AMI and were successful in identifying key lncRNAs involved in AMI [48–50], further emphasizing the potential of lncRNAs as biomarkers for the early diagnosis of AMI. However, the exact lncRNAs that hold this potential remain to be clarified.

Previous research suggested that the lncRNA *XIST* (X-inactive specific transcript, encoded by the *XIST* gene) is the cornerstone of mammalian X inactivation [51]. Based on accumulating evidence, the lncRNA *XIST* is important in genome maintenance, cell differentiation and proliferation [52, 53]. Liang et al. found that the downregulation of the lncRNA *XIST* and miR-7a-5P attenuate LPS-induced myocardial apoptosis in a mouse model of sepsis [54]. Similarly, Wang et al. described the involvement of the lncRNA XIST/miR-150-5p/c-Fos axis in LPS-induced myocardial injury, and the knockdown of the lncRNA *XIST* alleviates LPS-induced myocardial injury [55]. Peng et al. also found that the lncRNA *XIST* attenuates hypoxia-induced H9c2 cardiomyocyte injury by targeting the miR-122-5p/FOXP2 axis [56]. Furthermore, Zhang et al. proved that the silencing of the lncRNA *XIST* alleviates myocardial cell apoptosis in rats with AMI by targeting miR-449 [57]. According to Zhou et al., the lncRNA *XIST* is overexpressed in myocardial cells after MI and promotes MI by targeting miR-130a-3p [58]. In addition, Lin et al. also observed the overexpression of the lncRNA *XIST* in the infarct area and showed that the lncRNA *XIST* accelerated myocardial apoptosis after MI by targeting miR-101A-3p to upregulate FOS and apoptosis-related protein expression in a mouse model of MI [59]. Furthermore, Chen et al. found that lncRNA *XIST* can activate JAK2/STAT3 signal pathway by mediating mir-494/CDK6 regulatory axis, so as to promote the progression of esophageal cancer [60]. Zheng et al. found that silencing lncRNA *XIST* can effectively reduce the expression of *JAK2* by up-regulating Mir-337, thereby inhibiting the proliferation and migration of gastric cancer cells [61]. Although the above evidences suggest that *JAK2*, *CDC42* and lncRNA *XIST* are closely related to AMI, and lncRNA *XIST* can target and regulate the expression of *JAK2*. However, the aforementioned studies on the *JAK2*, *CDC42* and lncRNA *XIST* were all based on animal or cell models, and it is unclear whether lncRNA *XIST* is involved in AMI by regulating the expression of *JAK2* and *CDC42*. In order to illustrate this mechanism, our constructed a lncRNA-miRNA-mRNA triple network contained one lncRNA *XIST*, 21 miRNAs and 3 mRNAs (*JAK2*, *CDC42* and *CHUK*). We noticed that the expression of the *JAK2* and *CDC42* genes and the lncRNA *XIST* were all noticeably increased in patients with AMI compared to healthy subjects, and Pearson correlation analysis confirmed that the expression levels of *JAK2* and *CDC42* were positively correlated with the lncRNA *XIST* levels. Results from the ROC curve analysis further proved that the diagnostic efficacy of the lncRNA *XIST* was significantly better than that of *JAK2* and *CDC42*. Thus, we suggest that the lncRNA *XIST* may participate in the occurrence of AMI by targeting the expression of *JAK2* and *CDC42*, and lncRNA *XIST* may function as a reliable biomarker for the diagnosis of AMI.

This research has several limitations. First, the validation cohort included in the current study was recruited from only a single center and included small sample sizes. We did not clearly determine if variabilities exist variabilities among individuals from various regions and ethnicities. Therefore, the validity of the results in the current study must be further tested in multicenter and larger samples. Second, the miRNAs that participated in the lncRNA-miRNA-mRNA triple network not been verified in our validation samples. Follow-up experiments are needed to identify specific miRNAs that may be targeted by the lncRNA *XIST* to regulate *JAK2* and *CDC42* expression. Last, the specific mechanism by which the lncRNA-miRNA-mRNA network regulates the pathogenesis of AMI has not been verified *in vivo* and *in vitro*.

In conclusion, based on the ceRNA hypothesis, a triple regulatory network consisting of a lncRNA-miRNAs-mRNAs was constructed to explore its biological functions in AMI. We achieved this goal by analysing the gene expression profile of patients with AMI (GSE34198) using the WGCNA method. *JAK2*, *CDC42* and *CHUK* were identified as hub genes based on their combined GS and degree values. The identified regulatory network contained one lncRNA *XIST*, 21 miRNAs and 3 mRNAs (*JAK2*, *CDC42* and *CHUK*). We further identified the primary regulatory factors in this network by performing RT–qPCR combined with a ROC curve analysis and confirmed that the lncRNA *XIST* was involved in AMI, likely by modulating the expression of *JAK2* and *CDC42*, and lncRNA *XIST* may function as a reliable biomarker for the diagnosis of AMI.

## MATERIALS AND METHODS

### CAD microarray datasets

Gene expression data were extracted from the CAD dataset GSE34198 (including 49 AMI and 48 normal samples) in the public database Gene Expression Omnibus (GEO, http://www.ncbi.nlm.nih.gov/geo, which is hosted on the GPL6102 Illumina human-6 v2.0 expression BeadChip platform). Gene expression profiles were normalized using the normalize Between Arrays function in the limma package [62]. Probes that detected more than one gene were excluded from this study. The average expression level of the same gene detected with multiple different probes was calculated as the true expression level of the gene.

### Construction of the weighted gene co-expression network

A critical tool in the study of systems biology is WGCNA, which constructs a gene expression data profile-based scale-free network [63]. The reliability of the constructed scale-free network is ensured by removing outlying samples. A standard-scale free network was constructed to approximate the appropriate soft threshold power (soft power = 10) before the power function was used to calculate adjacency values among all differentially expressed genes. The adjacency values were converted into a topological overlap matrix (TOM), and the corresponding dissimilarity (1-TOM) values were also derived. The dynamic tree cut method was used to identify modules by hierarchically clustering genes with the 1-TOM as the distance measure, a minimum size cut-off of 100 and a deep split value of 2 for the resulting dendrogram. In addition, the module preservation function in the WGCNA package allowed us to determine the degree of module preservation and quality statistics [64]. These methods allowed us to verify the conservation of the selected modules.

### Preservation analysis of five network modules

Based on published analytical methods [65], the degree of conservativeness of 10 modules was assessed using a composite preservation statistics method based on the WGCNA R package modulePreservation function. Intramodular connectivity metrics and module density in each module were calculated using the Z-summary statistic. In the corresponding network, Zdensity (function 1) was used to calculate the 4 density preservation statistics, Zconnectivity (function 2) was used to calculate the 3 connectivity-based statistics, and the combined intramodular connectivity metrics and module density were measured using Zsummary (function 3) and defined as follows: Z _density_ = median (Z_meanCor_, Z_meanAdj_, Z_propVarExpl_, Z_meanKME_) (function 1); Z_connectivity_ = median (Z_cor.kIM_, Z_cor.kME_, Z_cor.cor_) (function 2); and Z_summary_ = (Z_density_ + Z_connectivity_)/2 (function 3). In addition, the module was not determined to be preserved if the Z summary < 2; modules were weakly to moderately preserved if the 2 < Z summary < 10; and modules were highly preserved if the Z summary > 10. Z statistics strongly depended on the size of the modules. Therefore, the medianRank for preservation analysis was used to assess preservation statistics between modules of various sizes. This analysis revealed that preservation statistics were more favorable in modules with a lower median rank in contrast to those with a higher median rank.

### Identification of the module of interest and functional annotation

The relationships between modules and clinical parameters were evaluated using Pearson’s correlation analysis to discern modules of biological significance. Gene Ontology (GO) and Kyoto Encyclopedia of Genes and Genomes (KEGG) enrichment analyses of genes in biologically significant modules were performed using the Database for Annotation, Visualization and Integrated Discovery (DAVID) online tool (version 6.8; https://david.ncifcrf.gov/). The threshold was set to *P* < 0.05.

### Hub gene analysis

The association between module eigengenes (Mes) and the gene expression profile was determined as the degree of module membership (MM). The absolute values of correlations between genes and external traits were defined as the degree of gene significance (GS). A further analysis of modules with increased GS and MM values was performed to determine biological functions [66]. Based on the meaningful modules, a protein–protein interaction (PPI) network was constructed using the Search Tool for the Retrieval of Interacting Genes (STRING) online tool (version 11.0; http://www.string-db.org) [67]. Cytoscape software was then used to visualize this PPI network [68]. Cytohubba, one of the most commonly used plug-ins in Cytoscape software, is often used to determine key genes in PPI networks [69]. This program is able to explore important nodes across various biological networks using 11 different methods, with the method of degree known to exhibit better performance. As reported in previous studies [65], genes with the top 50 GS values that also have degree values ≥ 5 were selected as key genes. Hub genes that required further research were also identified using information available from GO and KEGG analyses.

### The prediction of miRNAs and lincRNAs and ceRNA network construction

After GO, KEGG and PPI analyses, meaningful hub genes were used to predict lncRNAs that might regulate selected hub genes using the starBase database [70]. After obtaining the specific lncRNAs that may regulate the hub genes, lncRNA-miRNA interactions and miRNA-mRNA interactions were subsequently predicted based on the starBase database. A Venn diagram was constructed to identify several common miRNAs that potentially regulate both lncRNAs and mRNAs, and then the lncRNA-miRNA-mRNA ceRNA network was established and visualized using Cytoscape software.

### Study population

Five hundred inpatients (260 patients with AMI and 240 controls) with chest pain were recruited from the Cardiovascular Department of Hunan Provincial People's Hospital. All patients diagnosed with AMI underwent percutaneous coronary intervention (PCI) within 12 hours of the onset of chest pain. AMI was diagnosed based on 2018 guidelines for the diagnosis of patients with AMI [71] as follows: an electrocardiogram showing new ischemic changes, echocardiogram showing new localized ventricular wall dysplasia or a loss of viable myocardium, and cardiac biomarker (cTnT) levels above the upper limit of the reference value of the 99 percentile. Age- and sex-matched healthy subjects with no history of cardiovascular or other systemic diseases based on a physical examination, blood and electrocardiogram (ECG) tests were also recruited for this study. In addition, coronary angiography of all healthy subjects showed no significant abnormalities. The following exclusion criteria were applied: (i) active inflammation; (ii) patients who received thrombolytic therapy and those who had other underlying heart diseases (such as severe valvular abnormalities, cardiomyopathy, or congenital heart disease); and (iii) patients who had hepatic and/or renal dysfunction, tumours and autoimmune diseases. Baseline clinical characteristics, angiography results and laboratory test results were determined for all patients. Blood samples were collected from patients with AMI upon admission prior to the administration of any antiplatelet or anticoagulants, as well as before PCI treatment and within a few hours after the onset of chest pain. The collection of these samples was timed appropriately to capture the presence of any potential early diagnostic biomarkers. Study protocols were developed according to guidelines from the Ethics Committee of Hunan Provincial People's Hospital and the 2008 revision of the Declaration of Helsinki of 1975 (http://www.wma.net/en/30publications/10policies/b3/). All subjects provided written informed consent.

### RNA isolation and RT- quantitative PCR (qPCR)

TRIzol reagent (Invitrogen, CA, USA) was used to extract total RNA from all samples. The cDNA templates were then produced from 1 μg of RNA with the TransScript-Uni cDNA Synthesis SuperMix kit (AU311-03, Transgen, Beijing, China) with the GeneAmp PCR System 9700 HT Fast (Applied Biosystems, USA) for 60 min at 37° C. Real-time PCR was conducted with a LightCycler 480 II Real-time PCR instrument (Roche, Switzerland) using TransStart Top Green qPCR SuperMix (AQ131-03, Transgen, Beijing, China). At the end of amplification, the product quality was verified by constructing a melting curve. Samples were analysed in triplicate and included no-template controls. The quantitative RT-PCR analysis included 1 lncRNA X-inactive specific transcript (*XIST*) and 3 genes such as Janus kinase 2 (*JAK2*), cell division cycle 42 (*CDC42*) and component of inhibitor of nuclear factor kappa B kinase complex (*CHUK*). All gene expression levels were normalized to GAPDH. The proprietary qPCR primers used in the experiment are shown in Supplementary Table 1, and which were designed and validated by Songon Biotech (Songon Biotech, Shanghai, China). Relative gene expression was assessed with the 2^−ΔΔCt^ method.

### Statistical analyses

SPSS (Version 22.0) software was used to analyse all data collected in the current study. An independent sample t test was used to evaluate continuous data (means ± SD) that were normally distributed between control subjects and patients with AMI. Triglyceride levels that were not normally distributed were reported as medians and interquartile ranges and were evaluated using the Wilcoxon-Mann–Whitney test. Data such as the sex ratio and numbers of smokers and drinkers were analysed using the chi-square test. The nonparametric ROC curve analysis was performed using MedCalc software (MedCalc Software, Mariakerke, Belgium, version 19.7.4), which produced an empirical ROC curve and nonparametric estimate of the area under the empirical ROC curve along with its 95% CI, based on the method developed by Hanley et al. [72]. The difference between the areas under the two empirical ROC curves was assessed using the Z-test [73]. Bioinformatics analysis and Pearson’s correlation analysis were performed using R software (version 4.1.0).

## Supplementary Material

Supplementary Figure 1

Supplementary Table 1

Supplementary Table 2

Supplementary Table 3

Supplementary Table 4

Supplementary Table 5

Supplementary Table 6

Supplementary Table 7

Supplementary Table 8

Supplementary Table 9

Supplementary Table 10

Supplementary Table 11

Supplementary Table 12

## References

[1] Kishi S, Magalhães TA, Cerci RJ, Matheson MB, Vavere A, Tanami Y, Kitslaar PH, George RT, Brinker J, Miller JM, Clouse ME, Lemos PA, Niinuma H, et al. Total coronary atherosclerotic plaque burden assessment by CT angiography for detecting obstructive coronary artery disease associated with myocardial perfusion abnormalities. J Cardiovasc Comput Tomogr. 2016; 10:121–7. 10.1016/j.jcct.2016.01.005 26817414 PMC4788549

[2] Wong ND. Epidemiological studies of CHD and the evolution of preventive cardiology. Nat Rev Cardiol. 2014; 11:276–89. 10.1038/nrcardio.2014.26 24663092

[3] Xu H, Li W, Yang J, Wiviott SD, Sabatine MS, Peterson ED, Xian Y, Roe MT, Zhao W, Wang Y, Tang X, Jia X, Wu Y, et al, and CAMI Registry study group. The China Acute Myocardial Infarction (CAMI) Registry: A national long-term registry-research-education integrated platform for exploring acute myocardial infarction in China. Am Heart J. 2016; 175:193–201.e3. 10.1016/j.ahj.2015.04.014 27179740

[4] Chang J, Deng Q, Guo M, Ezzati M, Baumgartner J, Bixby H, Chan Q, Zhao D, Lu F, Hu P, Su Y, Sun J, Long Y, Liu J. Trends and Inequalities in the Incidence of Acute Myocardial Infarction among Beijing Townships, 2007-2018. Int J Environ Res Public Health. 2021; 18:12276. 10.3390/ijerph182312276 34886003 PMC8656834

[5] Khera AV, Kathiresan S. Genetics of coronary artery disease: discovery, biology and clinical translation. Nat Rev Genet. 2017; 18:331–44. 10.1038/nrg.2016.160 28286336 PMC5935119

[6] Haemmig S, Simion V, Yang D, Deng Y, Feinberg MW. Long noncoding RNAs in cardiovascular disease, diagnosis, and therapy. Curr Opin Cardiol. 2017; 32:776–83. 10.1097/HCO.0000000000000454 28786864 PMC5892448

[7] Osmak G, Baulina N, Koshkin P, Favorova O. Collapsing the list of myocardial infarction-related differentially expressed genes into a diagnostic signature. J Transl Med. 2020; 18:231. 10.1186/s12967-020-02400-1 32517814 PMC7285786

[8] Aryal B, Rotllan N, Fernández-Hernando C. Noncoding RNAs and atherosclerosis. Curr Atheroscler Rep. 2014; 16:407. 10.1007/s11883-014-0407-3 24623179 PMC4145585

[9] Fu XD. Non-coding RNA: a new frontier in regulatory biology. Natl Sci Rev. 2014; 1:190–204. 10.1093/nsr/nwu008 25821635 PMC4374487

[10] Boon RA, Jaé N, Holdt L, Dimmeler S. Long Noncoding RNAs: From Clinical Genetics to Therapeutic Targets? J Am Coll Cardiol. 2016; 67:1214–26. 10.1016/j.jacc.2015.12.051 26965544

[11] Esteller M. Non-coding RNAs in human disease. Nat Rev Genet. 2011; 12:861–74. 10.1038/nrg3074 22094949

[12] Wang KC, Chang HY. Molecular mechanisms of long noncoding RNAs. Mol Cell. 2011; 43:904–14. 10.1016/j.molcel.2011.08.018 21925379 PMC3199020

[13] Quinn JJ, Chang HY. Unique features of long non-coding RNA biogenesis and function. Nat Rev Genet. 2016; 17:47–62. 10.1038/nrg.2015.10 26666209

[14] Correia CCM, Rodrigues LF, de Avila Pelozin BR, Oliveira EM, Fernandes T. Long Non-Coding RNAs in Cardiovascular Diseases: Potential Function as Biomarkers and Therapeutic Targets of Exercise Training. Noncoding RNA. 2021; 7:65. 10.3390/ncrna7040065 34698215 PMC8544698

[15] Wang K, Long B, Zhou LY, Liu F, Zhou QY, Liu CY, Fan YY, Li PF. CARL lncRNA inhibits anoxia-induced mitochondrial fission and apoptosis in cardiomyocytes by impairing miR-539-dependent PHB2 downregulation. Nat Commun. 2014; 5:3596. 10.1038/ncomms4596 24710105

[16] Micheletti R, Plaisance I, Abraham BJ, Sarre A, Ting CC, Alexanian M, Maric D, Maison D, Nemir M, Young RA, Schroen B, González A, Ounzain S, Pedrazzini T. The long noncoding RNA Wisper controls cardiac fibrosis and remodeling. Sci Transl Med. 2017; 9:eaai9118. 10.1126/scitranslmed.aai9118 28637928 PMC5643582

[17] Huang Y. The novel regulatory role of lncRNA-miRNA-mRNA axis in cardiovascular diseases. J Cell Mol Med. 2018; 22:5768–75. 10.1111/jcmm.13866 30188595 PMC6237607

[18] Ratti M, Lampis A, Ghidini M, Salati M, Mirchev MB, Valeri N, Hahne JC. MicroRNAs (miRNAs) and Long Non-Coding RNAs (lncRNAs) as New Tools for Cancer Therapy: First Steps from Bench to Bedside. Target Oncol. 2020; 15:261–78. 10.1007/s11523-020-00717-x 32451752 PMC7283209

[19] Miao L, Yin RX, Pan SL, Yang S, Yang DZ, Lin WX. Weighted Gene Co-Expression Network Analysis Identifies Specific Modules and Hub Genes Related to Hyperlipidemia. Cell Physiol Biochem. 2018; 48:1151–63. 10.1159/000491982 30045016

[20] Chen ZH, Zhang M, Li YC, Zhao ZP, Zhang X, Huang ZJ, Li C, Wang LM. [Study on relationship between prevalence or co-prevalence of risk factors for cardiovascular disease and blood pressure level in adults in China]. Zhonghua Liu Xing Bing Xue Za Zhi. 2018; 39:640–5. 10.3760/cma.j.issn.0254-6450.2018.05.019 29860809

[21] Xiao SJ, Zhou YF, Wu Q, Ma WR, Chen ML, Pan DF. Uncovering the differentially expressed genes and pathways involved in the progression of stable coronary artery disease to acute myocardial infarction using bioinformatics analysis. Eur Rev Med Pharmacol Sci. 2021; 25:301–12. 10.26355/eurrev_202101_24396 33506919

[22] Zhang QH, Yin RX, Chen WX, Cao XL, Wu JZ. TRIB1 and TRPS1 variants, G × G and G × E interactions on serum lipid levels, the risk of coronary heart disease and ischemic stroke. Sci Rep. 2019; 9:2376. 10.1038/s41598-019-38765-7 30787327 PMC6382757

[23] Zheng PF, Yin RX, Deng GX, Guan YZ, Wei BL, Liu CX. Association between the XKR6 rs7819412 SNP and serum lipid levels and the risk of coronary artery disease and ischemic stroke. BMC Cardiovasc Disord. 2019; 19:202. 10.1186/s12872-019-1179-z 31429711 PMC6700994

[24] Libby P, Theroux P. Pathophysiology of coronary artery disease. Circulation. 2005; 111:3481–8. 10.1161/CIRCULATIONAHA.105.537878 15983262

[25] Li B, Li W, Li X, Zhou H. Inflammation: A Novel Therapeutic Target/Direction in Atherosclerosis. Curr Pharm Des. 2017; 23:1216–27. 10.2174/1381612822666161230142931 28034355 PMC6302344

[26] Zheng PF, Chen LZ, Guan YZ, Liu P. Weighted gene co-expression network analysis identifies specific modules and hub genes related to coronary artery disease. Sci Rep. 2021; 11:6711. 10.1038/s41598-021-86207-0 33758323 PMC7988178

[27] Liao M, Xu J, Clair AJ, Ehrman B, Graham LM, Eagleton MJ. Local and systemic alterations in signal transducers and activators of transcription (STAT) associated with human abdominal aortic aneurysms. J Surg Res. 2012; 176:321–8. 10.1016/j.jss.2011.05.041 21764069 PMC3197955

[28] Manea A, Tanase LI, Raicu M, Simionescu M. Jak/STAT signaling pathway regulates nox1 and nox4-based NADPH oxidase in human aortic smooth muscle cells. Arterioscler Thromb Vasc Biol. 2010; 30:105–12. 10.1161/ATVBAHA.109.193896 19834108

[29] Mo ZC, Xiao J, Liu XH, Hu YW, Li XX, Yi GH, Wang Z, Tang YL, Liao DF, Tang CK. AOPPs inhibits cholesterol efflux by down-regulating ABCA1 expression in a JAK/STAT signaling pathway-dependent manner. J Atheroscler Thromb. 2011; 18:796–807. 10.5551/jat.6569 21670559

[30] Yang X, Jia J, Yu Z, Duanmu Z, He H, Chen S, Qu C. Inhibition of JAK2/STAT3/SOCS3 signaling attenuates atherosclerosis in rabbit. BMC Cardiovasc Disord. 2020; 20:133. 10.1186/s12872-020-01391-7 32169038 PMC7071770

[31] Zhang X, Li J, Qin JJ, Cheng WL, Zhu X, Gong FH, She Z, Huang Z, Xia H, Li H. Oncostatin M receptor β deficiency attenuates atherogenesis by inhibiting JAK2/STAT3 signaling in macrophages. J Lipid Res. 2017; 58:895–906. 10.1194/jlr.M074112 28258089 PMC5408608

[32] Desai HR, Sivasubramaniyam T, Revelo XS, Schroer SA, Luk CT, Rikkala PR, Metherel AH, Dodington DW, Park YJ, Kim MJ, Rapps JA, Besla R, Robbins CS, et al. Macrophage JAK2 deficiency protects against high-fat diet-induced inflammation. Sci Rep. 2017; 7:7653. 10.1038/s41598-017-07923-0 28794431 PMC5550513

[33] Geng Z, Fan WY, Zhou B, Ye C, Tong Y, Zhou YB, Xiong XQ. FNDC5 attenuates obesity-induced cardiac hypertrophy by inactivating JAK2/STAT3-associated inflammation and oxidative stress. J Transl Med. 2019; 17:107. 10.1186/s12967-019-1857-8 30940158 PMC6444535

[34] Zhang Y, Li J, Lai XN, Jiao XQ, Xiong JP, Xiong LX. Focus on Cdc42 in Breast Cancer: New Insights, Target Therapy Development and Non-Coding RNAs. Cells. 2019; 8:146. 10.3390/cells8020146 30754684 PMC6406589

[35] Huang QY, Lai XN, Qian XL, Lv LC, Li J, Duan J, Xiao XH, Xiong LX. Cdc42: A Novel Regulator of Insulin Secretion and Diabetes-Associated Diseases. Int J Mol Sci. 2019; 20:179. 10.3390/ijms20010179 30621321 PMC6337499

[36] Ito TK, Yokoyama M, Yoshida Y, Nojima A, Kassai H, Oishi K, Okada S, Kinoshita D, Kobayashi Y, Fruttiger M, Aiba A, Minamino T. A crucial role for CDC42 in senescence-associated inflammation and atherosclerosis. PLoS One. 2014; 9:e102186. 10.1371/journal.pone.0102186 25057989 PMC4109913

[37] Raut SK, Kumar A, Singh GB, Nahar U, Sharma V, Mittal A, Sharma R, Khullar M. miR-30c Mediates Upregulation of Cdc42 and Pak1 in Diabetic Cardiomyopathy. Cardiovasc Ther. 2015; 33:89–97. 10.1111/1755-5922.12113 25781190

[38] Ye H, Ling S, Castillo AC, Thomas B, Long B, Qian J, Perez-Polo JR, Ye Y, Chen X, Birnbaum Y. Nebivolol induces distinct changes in profibrosis microRNA expression compared with atenolol, in salt-sensitive hypertensive rats. Hypertension. 2013; 61:1008–13. 10.1161/HYPERTENSIONAHA.111.00892 23460283

[39] Liu D, Tian X, Liu Y, Song H, Cheng X, Zhang X, Yan C, Han Y. CREG ameliorates the phenotypic switching of cardiac fibroblasts after myocardial infarction via modulation of CDC42. Cell Death Dis. 2021; 12:355. 10.1038/s41419-021-03623-w 33824277 PMC8024263

[40] Li X, Wu Z, Fu X, Han W. lncRNAs: insights into their function and mechanics in underlying disorders. Mutat Res Rev Mutat Res. 2014; 762:1–21. 10.1016/j.mrrev.2014.04.002 25485593

[41] Li M, Wang YF, Yang XC, Xu L, Li WM, Xia K, Zhang DP, Wu RN, Gan T. Circulating Long Noncoding RNA LIPCAR Acts as a Novel Biomarker in Patients with ST-Segment Elevation Myocardial Infarction. Med Sci Monit. 2018; 24:5064–70. 10.12659/MSM.909348 30030914 PMC6067052

[42] Iyer MK, Niknafs YS, Malik R, Singhal U, Sahu A, Hosono Y, Barrette TR, Prensner JR, Evans JR, Zhao S, Poliakov A, Cao X, Dhanasekaran SM, et al. The landscape of long noncoding RNAs in the human transcriptome. Nat Genet. 2015; 47:199–208. 10.1038/ng.3192 25599403 PMC4417758

[43] Ørom UA, Derrien T, Beringer M, Gumireddy K, Gardini A, Bussotti G, Lai F, Zytnicki M, Notredame C, Huang Q, Guigo R, Shiekhattar R. Long noncoding RNAs with enhancer-like function in human cells. Cell. 2010; 143:46–58. 10.1016/j.cell.2010.09.001 20887892 PMC4108080

[44] Ounzain S, Micheletti R, Beckmann T, Schroen B, Alexanian M, Pezzuto I, Crippa S, Nemir M, Sarre A, Johnson R, Dauvillier J, Burdet F, Ibberson M, et al. Genome-wide profiling of the cardiac transcriptome after myocardial infarction identifies novel heart-specific long non-coding RNAs. Eur Heart J. 2015; 36:353–68a. 10.1093/eurheartj/ehu180 24786300 PMC4320320

[45] Wang K, Liu F, Zhou LY, Long B, Yuan SM, Wang Y, Liu CY, Sun T, Zhang XJ, Li PF. The long noncoding RNA CHRF regulates cardiac hypertrophy by targeting miR-489. Circ Res. 2014; 114:1377–88. 10.1161/CIRCRESAHA.114.302476 24557880

[46] Zangrando J, Zhang L, Vausort M, Maskali F, Marie PY, Wagner DR, Devaux Y. Identification of candidate long non-coding RNAs in response to myocardial infarction. BMC Genomics. 2014; 15:460. 10.1186/1471-2164-15-460 24917243 PMC4070571

[47] Qu X, Song X, Yuan W, Shu Y, Wang Y, Zhao X, Gao M, Lu R, Luo S, Zhao W, Zhang Y, Sun L, Lu Y. Expression signature of lncRNAs and their potential roles in cardiac fibrosis of post-infarct mice. Biosci Rep. 2016; 36:e00337. 10.1042/BSR20150278 27129287 PMC5293569

[48] Sun C, Jiang H, Sun Z, Gui Y, Xia H. Identification of long non-coding RNAs biomarkers for early diagnosis of myocardial infarction from the dysregulated coding-non-coding co-expression network. Oncotarget. 2016; 7:73541–51. 10.18632/oncotarget.11999 27634901 PMC5341997

[49] Wang P, Fu H, Cui J, Chen X. Differential lncRNA-mRNA co-expression network analysis revealing the potential regulatory roles of lncRNAs in myocardial infarction. Mol Med Rep. 2016; 13:1195–203. 10.3892/mmr.2015.4669 26676325 PMC4732855

[50] Zhuo LA, Wen YT, Wang Y, Liang ZF, Wu G, Nong MD, Miao L. LncRNA SNHG8 is identified as a key regulator of acute myocardial infarction by RNA-seq analysis. Lipids Health Dis. 2019; 18:201. 10.1186/s12944-019-1142-0 31739782 PMC6862811

[51] Brown CJ, Ballabio A, Rupert JL, Lafreniere RG, Grompe M, Tonlorenzi R, Willard HF. A gene from the region of the human X inactivation centre is expressed exclusively from the inactive X chromosome. Nature. 1991; 349:38–44. 10.1038/349038a0 1985261

[52] Yildirim E, Kirby JE, Brown DE, Mercier FE, Sadreyev RI, Scadden DT, Lee JT. Xist RNA is a potent suppressor of hematologic cancer in mice. Cell. 2013; 152:727–42. 10.1016/j.cell.2013.01.034 23415223 PMC3875356

[53] Yao Y, Ma J, Xue Y, Wang P, Li Z, Liu J, Chen L, Xi Z, Teng H, Wang Z, Li Z, Liu Y. Knockdown of long non-coding RNA XIST exerts tumor-suppressive functions in human glioblastoma stem cells by up-regulating miR-152. Cancer Lett. 2015; 359:75–86. 10.1016/j.canlet.2014.12.051 25578780

[54] Liang D, Jin Y, Lin M, Xia X, Chen X, Huang A. Down-regulation of Xist and Mir-7a-5p improves LPS-induced myocardial injury. Int J Med Sci. 2020; 17:2570–7. 10.7150/ijms.45408 33029099 PMC7532474

[55] Wang X, Li XL, Qin LJ. The lncRNA XIST/miR-150-5p/c-Fos axis regulates sepsis-induced myocardial injury via TXNIP-modulated pyroptosis. Lab Invest. 2021; 101:1118–29. 10.1038/s41374-021-00607-4 34045679

[56] Peng H, Luo Y, Ying Y. lncRNA XIST attenuates hypoxia-induced H9c2 cardiomyocyte injury by targeting the miR-122-5p/FOXP2 axis. Mol Cell Probes. 2020; 50:101500. 10.1016/j.mcp.2019.101500 31887421

[57] Zhang M, Liu HY, Han YL, Wang L, Zhai DD, Ma T, Zhang MJ, Liang CZ, Shen Y. Silence of lncRNA XIST represses myocardial cell apoptosis in rats with acute myocardial infarction through regulating miR-449. Eur Rev Med Pharmacol Sci. 2019; 23:8566–72. 10.26355/eurrev_201910_19172 31646589

[58] Zhou T, Qin G, Yang L, Xiang D, Li S. LncRNA XIST regulates myocardial infarction by targeting miR-130a-3p. J Cell Physiol. 2019; 234:8659–67. 10.1002/jcp.26327 29226319

[59] Lin B, Xu J, Wang F, Wang J, Zhao H, Feng D. LncRNA XIST promotes myocardial infarction by regulating FOS through targeting miR-101a-3p. Aging (Albany NY). 2020; 12:7232–47. 10.18632/aging.103072 32315985 PMC7202499

[60] Chen Z, Hu X, Wu Y, Cong L, He X, Lu J, Feng J, Liu D. Long non-coding RNA XIST promotes the development of esophageal cancer by sponging miR-494 to regulate CDK6 expression. Biomed Pharmacother. 2019; 109:2228–36. 10.1016/j.biopha.2018.11.049 30551480

[61] Zheng W, Li J, Zhou X, Cui L, Wang Y. The lncRNA XIST promotes proliferation, migration and invasion of gastric cancer cells by targeting miR-337. Arab J Gastroenterol. 2020; 21:199–206. 10.1016/j.ajg.2020.07.010 32830093

[62] Ritchie ME, Phipson B, Wu D, Hu Y, Law CW, Shi W, Smyth GK. limma powers differential expression analyses for RNA-sequencing and microarray studies. Nucleic Acids Res. 2015; 43:e47. 10.1093/nar/gkv007 25605792 PMC4402510

[63] Horvath S, Dong J. Geometric interpretation of gene coexpression network analysis. PLoS Comput Biol. 2008; 4:e1000117. 10.1371/journal.pcbi.1000117 18704157 PMC2446438

[64] Langfelder P, Horvath S. WGCNA: an R package for weighted correlation network analysis. BMC Bioinformatics. 2008; 9:559. 10.1186/1471-2105-9-559 19114008 PMC2631488

[65] Chen J, Zhao X, Cui L, He G, Wang X, Wang F, Duan S, He L, Li Q, Yu X, Zhang F, Xu M. Genetic regulatory subnetworks and key regulating genes in rat hippocampus perturbed by prenatal malnutrition: implications for major brain disorders. Aging (Albany NY). 2020; 12:8434–58. 10.18632/aging.103150 32392183 PMC7244046

[66] Fuller TF, Ghazalpour A, Aten JE, Drake TA, Lusis AJ, Horvath S. Weighted gene coexpression network analysis strategies applied to mouse weight. Mamm Genome. 2007; 18:463–72. 10.1007/s00335-007-9043-3 17668265 PMC1998880

[67] Szklarczyk D, Franceschini A, Wyder S, Forslund K, Heller D, Huerta-Cepas J, Simonovic M, Roth A, Santos A, Tsafou KP, Kuhn M, Bork P, Jensen LJ, von Mering C. STRING v10: protein-protein interaction networks, integrated over the tree of life. Nucleic Acids Res. 2015; 43:D447–52. 10.1093/nar/gku1003 25352553 PMC4383874

[68] Smoot ME, Ono K, Ruscheinski J, Wang PL, Ideker T. Cytoscape 2.8: new features for data integration and network visualization. Bioinformatics. 2011; 27:431–2. 10.1093/bioinformatics/btq675 21149340 PMC3031041

[69] Chin CH, Chen SH, Wu HH, Ho CW, Ko MT, Lin CY. cytoHubba: identifying hub objects and sub-networks from complex interactome. BMC Syst Biol. 2014 (Suppl 4); 8:S11. 10.1186/1752-0509-8-S4-S11 25521941 PMC4290687

[70] Li JH, Liu S, Zhou H, Qu LH, Yang JH. starBase v2.0: decoding miRNA-ceRNA, miRNA-ncRNA and protein-RNA interaction networks from large-scale CLIP-Seq data. Nucleic Acids Res. 2014; 42:D92–7. 10.1093/nar/gkt1248 24297251 PMC3964941

[71] Thygesen K, Alpert JS, Jaffe AS, Chaitman BR, Bax JJ, Morrow DA, White HD, and Executive Group on behalf of the Joint European Society of Cardiology (ESC)/American College of Cardiology (ACC)/American Heart Association (AHA)/World Heart Federation (WHF) Task Force for the Universal Definition of Myocardial Infarction. Fourth Universal Definition of Myocardial Infarction (2018). Circulation. 2018; 138:e618–51. 10.1161/CIR.0000000000000617 30571511

[72] Hanley JA, McNeil BJ. The meaning and use of the area under a receiver operating characteristic (ROC) curve. Radiology. 1982; 143:29–36. 10.1148/radiology.143.1.7063747 7063747

[73] Hanley JA, McNeil BJ. A method of comparing the areas under receiver operating characteristic curves derived from the same cases. Radiology. 1983; 148:839–43. 10.1148/radiology.148.3.6878708 6878708

