# Supplementary material for: A novel lncRNA-miRNA-mRNA triple network identifies lncRNA XIST as a biomarker for acute myocardial infarction
Source: Aging (Albany NY). 2022 May 10;14(9):4085–106. doi: 10.18632/aging.204075 (PMC9134965; doi:10.18632/aging.204075)
Supplement: Supplementary Figure 1 [file aging-14-204075-s001.pdf]

SUPPLEMENTARY FIGURE

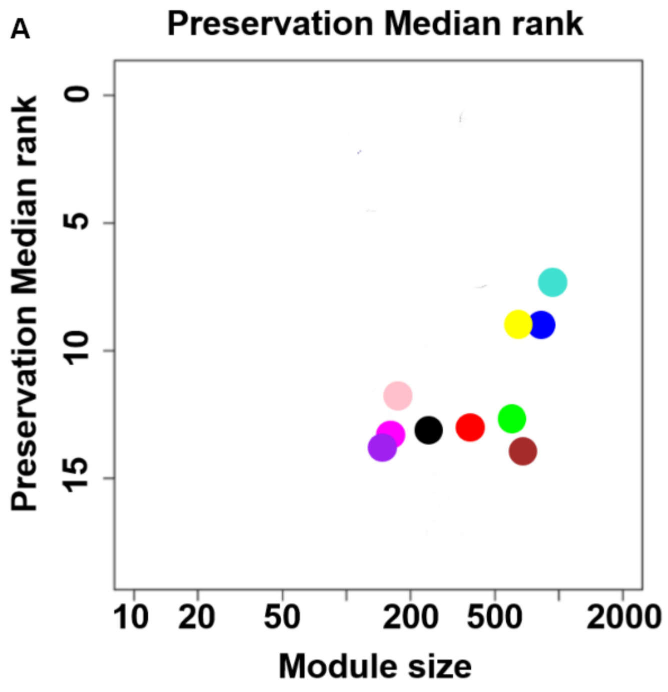

Supplementary Figure 1. Preservation analysis of five network modules. The Y-axis represents preserved values and the X-axis represents module size. (**A**) median Rank test; and (**B**) Z summary statistics test.
