# Supplementary material for: A novel lncRNA-miRNA-mRNA triple network identifies lncRNA XIST as a biomarker for acute myocardial infarction
Source: Aging (Albany NY). 2022 May 10;14(9):4085–106. doi: 10.18632/aging.204075 (PMC9134965; doi:10.18632/aging.204075)
Supplement: Supplementary Table 1 [file aging-14-204075-s002.pdf]

SUPPLEMENTARY TABLE

Supplementary Table 1. PCR primers for quantitative real-time PCR.

Gene	Forward primer	Reverse primer
XIST	5′-CAGACGTGTGCTCTTC-3′	5′-CGATCTGTAAGTCCACCA-3′
JAK2	5′-TCTGGGGAGTATGTTGCAGAA-3′	5′-AGACATGGTTGGGTGGATACC-3′
CHUK	5′-GCATCATCTGCAGCCATTTA-3′	5′-CAACAGGTCCTCCTCTCTGC-3′
CDC42	5′-GAAGGCTGTCAAGTATGTGG-3′	5′-CTCTTCTTCGGTTCTGGAGG-3′
GAPDH	5′-ACCCAGAAGACTGTGGATGG-3′	5′-CACATTGGGGGTAGGAACAC-3′
