# Supplementary material for: A novel lncRNA-miRNA-mRNA triple network identifies lncRNA XIST as a biomarker for acute myocardial infarction
Source: Aging (Albany NY). 2022 May 10;14(9):4085–106. doi: 10.18632/aging.204075 (PMC9134965; doi:10.18632/aging.204075)
Supplement: Supplementary Table 8 [file aging-14-204075-s009.pdf]

Supplementary Table 8. Total of 27 genes with the top 50 GS values and degree values ≥ 5.

GS-PPI common genes	Degree	Gene significance	p.GS
CDC42	64	0.376966277	0.000141252
JAK2	41	0.386232529	0.0000933
CHUK	30	0.397403719	0.0000556
EPS15	28	0.356581164	0.000337089
TBK1	25	0.34480493	0.000542962
CMTM6	21	0.365867874	0.000228432
UBE2W	20	0.34129215	0.000623711
STX7	19	0.35431781	0.000369963
RB1CC1	18	0.351771236	0.000410461
GNAI3	18	0.339381638	0.000672107
RHOT1	18	0.389460613	0.0000805
USP15	16	0.3512465	0.000419297
NT5C2	15	0.412844637	0.0000264
GCA	14	0.352973892	0.000390853
RAB21	14	0.340633205	0.00064003
PGM2	13	0.362010643	0.000268886
CHMP2B	13	0.348314938	0.000471956
DCP2	11	0.349822612	0.000444159
MAP3K2	10	0.349625256	0.00044771
TANK	10	0.392836595	0.0000689
RPGR	10	0.343529475	0.000571102
SCLT1	7	0.449967423	0.00000376
OTUD1	6	0.348341685	0.000471449
SLK	6	0.34532133	0.000531935
BLZF1	6	0.358564592	0.000310514
SNX10	5	0.346331928	0.000510947
SELT	5	0.339541077	0.000667941
