# Supplementary material for: A novel lncRNA-miRNA-mRNA triple network identifies lncRNA XIST as a biomarker for acute myocardial infarction
Source: Aging (Albany NY). 2022 May 10;14(9):4085–106. doi: 10.18632/aging.204075 (PMC9134965; doi:10.18632/aging.204075)
Supplement: Supplementary Table 12 [file aging-14-204075-s013.pdf]

Supplementary Table 12. All of miRNAs that can simultaneously target JAK2, CDC42, CHUK and lncRNA XIST.

XIST-CDC42-CHUK-JAK2 common miRNAs
hsa-miR-381-3p
hsa-miR-92b-3p
hsa-miR-195-5p
hsa-miR-497-5p
hsa-miR-424-5p
hsa-miR-363-3p
hsa-miR-15b-5p
hsa-miR-380-3p
hsa-miR-32-5p
hsa-miR-105-5p
hsa-miR-524-5p
hsa-miR-92a-3p
hsa-miR-377-3p
hsa-miR-300
hsa-miR-25-3p
hsa-miR-367-3p
hsa-miR-15a-5p
hsa-miR-410-3p
hsa-miR-214-3p
hsa-miR-520d-5p
hsa-miR-16-5p
